# Supplementary material for: Temporal trends of cutaneo-mucous histoplasmosis in persons living with HIV in French Guiana: Early diagnosis defuses South American strain dermotropism
Source: PLoS Negl Trop Dis. 2020 Oct 19;14(10):e0008663. doi: 10.1371/journal.pntd.0008663 (PMC7595617; doi:10.1371/journal.pntd.0008663)
Supplement: Attachment — Submitted filename: plosntds revision letter.docx [file pntd.0008663.s001.docx]

*Dear Editor, we are grateful for the reviewer comments which have helped us improve the manuscript. You will find the responses hereunder; we hope you will find them satisfactory, and that you will agree that the manuscript has significantly improved.*

Reviewer #1: The methods are adequate despite the limitation of every retrospective study.

Reviewer #2: The study design does not seem adequate to the proposed objectives. Incidence cannot be estimated with a cross-sectional study.

*=>thank you for raising this point in fact the study is not cross sectional. It was the retrospective analysis of the HIV cohort (French Hospital Database on HIV (FHDH) which continually collects patient data, incident infections, events. Our analysis of this dataset aimed to look at the historical perspective which is a “macro” approach we thought had its virtues regarding the hypothesis at hand.*

The authors demonstrate that they have a valuable database, but that it should be better explored. This imperfection is critical and, therefore, the whole text should be rewritten. The wrong choice of a study design ends up causing obstacles to the data analysis.
There is a lack of information regarding the clinical presentation of histoplasmosis, like measuring the difference between the onset of symptoms and the date of diagnosis.

*=> unfortunately this interesting information is not a variable that is present in the database.*

The same applies to findings regarding HIV infection. Information such as CD4 nadir, course of HIV infection, and year of HIV diagnosis. Understand that the diagnosis of an opportunistic infection may have a different interpretation if it occurred before or after the HAART policy introduction. It may reflect the high occurrence of late diagnoses, low adherence to HAART, or resistance to HAART in the sample. It is also imperative to analyze histoplasmosis incidence based on the prevalence of HIV infection in the pre and post HAART periods.

# *=> Indeed it is important information. the estimation of prevalence is based on models using spectrum software (avenir health) that have been published recently (Modeling of the HIV epidemic and continuum of care in French Guiana)* [*https://doi.org/10.1371/journal.pone.0197990*](https://doi.org/10.1371/journal.pone.0197990)*. Here we added a graph constructed by collapsing the database by year showing the evolution of the number of new diagnoses and the median CD4 count at the time of diagnosis. What we see is the rise in the 1990s and after that a greater proportion of immunocompromised patients. What this adds is that although the median CD4 count was higher the proportion of cutaneous and mucous lesions was higher suggesting that diagnostic capacity improved over time.*

Reviewer #1: The manuscript “Temporal trends of cutaneo-mucous histoplasmosis in persons living with HIV in French Guiana: early diagnosis defuses South American strain dermotropism” by Morote et al, investigates a deeply-discussed topic regarding the clinical manifestation of HIV-associated histoplasmosis between USA and Latin America. The manuscript and the retrospective study (despite the obvious limitations) are well presented and designed and should be considered for publication. There are few minor observations:

Introduction

Histoplasmosis – do not capitalize

*=>ok*
Histoplasma – Italicize

*=>ok*
“In contrast, looking at the historical perspective of clinical presentation allows to vary access to diagnosis assuming” – Does not read well…please clarify “allows to vary access”
*=>we agree it is poorly written. We hope this formulation is better “In contrast, looking at the historical perspective of clinical presentation allows to study its changes as access to diagnosis improved over time assuming that endemic Histoplasma isolates remained genetically similar.”*

Methods

“all patients living with HIV known” – HIV-living patients?

*=> again the formulation was not clear we have changed for known HIV*

Discussion

There is a lack of discussion regarding the genetic background of the players between North and Latin America: The disease in United Stated are caused by H. mississipiensis and H. ohiensis while the disease in Latin America is caused by H. capsulatum and H. suramericanum (See Sepulveda et al. MBio 2017). Prospective studies aiming to investigate the genetic background on the disease presentations are needed.

*=>ok we have specified thank you for the suggestions*

How this trend in of cutaneo-mucous histoplasmosis presentations is comparable to other fungal infections caused by Onygenales (i.e.: paracoccidioidomycosis and emmergomycosis) in HIV patients? Late diagnosis in other mycoses caused by dimorphic fungi can lead to skin disseminated disease?

*=>indeed this is interesting but in French Guiana we rarely see paracoccidioidomycosis and so far we have not seen emmergomycosis perhaps authors from endemic areas will see patterns emerging.*

Reviewer #2: Please, note that tuberculosis, with a microbiologically confirmed diagnosis, is definitely the most common opportunistic infection related to AIDS in Latin America. It cannot be valid in French Guiana but must be addressed in the text.
*=>in French Guiana Histoplasmosis is the main opportunistic every year and first AIDS-related cause of death*. *The reference by Adenis modelled this using paho data for tuberculosis and found that in some countries it is tuberculosis that is number one and in many other countries it is histoplasmosis. Not to minimize the massive importance of tuberculosis in persons with HIV, histoplasmosis is also a very important topic. We have rephrased to be less sweeping in the description of the modelling study.*
